# Digital twins as run-time predictive models for the resilience of cyber-physical systems: a conceptual framework

**DOI:** 10.1098/rsta.2020.0369

**Published:** 2021-10-04

**Authors:** Francesco Flammini

**Affiliations:** School of Design, Engineering and Technology, Mälardalen University, Hamngatan 15, 632 20 Eskilstuna, Sweden

**Keywords:** digital twins, trustworthy autonomy, cyber-physical systems, run-time models, self-healing, resilience

## Abstract

Digital twins (DT) are emerging as an extremely promising paradigm for run-time modelling and performability prediction of cyber-physical systems (CPS) in various domains. Although several different definitions and industrial applications of DT exist, ranging from purely visual three-dimensional models to predictive maintenance tools, in this paper, we focus on data-driven evaluation and prediction of critical dependability attributes such as safety. To that end, we introduce a conceptual framework based on autonomic systems to host DT run-time models based on a structured and systematic approach. We argue that the convergence between DT and self-adaptation is the key to building smarter, resilient and trustworthy CPS that can self-monitor, self-diagnose and—ultimately—self-heal. The conceptual framework eases dependability assessment, which is essential for the certification of autonomous CPS operating with artificial intelligence and machine learning in critical applications.

This article is part of the theme issue ‘Towards symbiotic autonomous systems’.

## 1. Introduction

Critical computer-based systems, including cyber-physical systems (CPS) and the Internet of Things (IoT), combining both tangible and virtual entities, permeate our everyday lives in modern society and are therefore becoming increasingly symbiotic with humans [1]. Criticality refers to the effects of malfunctions and failures, which can have consequences ranging from the loss of money (e.g. financial frauds through cyber-threats) to the loss of human lives (e.g. accidents in intelligent transportation systems). Therefore, those systems need to be accurately designed and monitored in order to reduce the risk of malfunctions [2], possibly using agent-based approaches [3,4]. A class of those systems also needs to be certified against international well-established reliability, safety and security standards [5,6].

While predictability was the key for the assessment of legacy systems such as trains or airplanes, nowadays there is a paradigm shift toward smarter systems based on artificial intelligence (AI), which have the advantage of learning and adapting to new situations; however, those characteristics also introduce a high level of uncertainty that complicates their analysis and certification [7]. Therefore, we are witnessing an apparent paradox where systems have the potential of becoming more dependable due to their higher intelligence, but that can also reduce the level of trust we hold in those systems. Complexity itself, as a result of growing size, distribution and heterogeneity, is another obstacle to full predictability.

In such a scenario, run-time models represent a viable option to manage dependability aspects in the operational stage through perpetual assurances. In other words, parts of the assessment models remain alive from design-time to run-time, and that allows us to deal with certain classes of uncertainties and changes, both in the system and in the environment in which it operates [8]. This is where the paradigms of digital twin (DT) and autonomic computing can help by providing an effective conceptual model to frame the theoretical and practical aspects related to how to manage run-time models in terms of data collection, model execution, explainability, planning of reconfigurations and implementation of response actions.

In this paper, we provide a conceptual, high-level framework that summarizes recent research and future opportunities in using DT as run-time predictive models for resilience, self-healing and trustworthy autonomy of CPS. While some ideas are being investigated for using DT in infrastructure resilience applications (see, e.g. reference [9]), to the best of our knowledge, the concept of integrating DT and autonomic computing into a single conceptual framework has not been addressed yet by the scientific community. The only work that recently discussed some opportunities in this field is reported in reference [10]; however, in that work, the authors mainly addressed a case study of model-driven engineering connected to reflective architectural patterns, without generalizing the DT approach to CPS architectures at multiple abstraction levels. Further to such generalization, based on the theory of autonomic computing for self-healing, in this paper, we also provide some hints about anomaly detection with process mining through holistic approaches that are suitable to the assessment of cooperative IoT systems-of-systems [11].

The rest of this paper is structured as follows. Section 2 provides a brief summary of basic concepts and current research areas in the field of CPS resilience, self-healing and trustworthy autonomy. Section 3 introduces our conceptual framework and reference architecture where a convergence is sought between the concepts of DT and autonomic computing, with a focus on CPS resilience. Section 4 provides an overview of the challenges of detecting behavioural anomalies in distributed CPS in order to inspire further research directions. Finally, §5 will draw conclusions and provide some hints about future developments.

## 2. From cyber-physical system resilience to trustworthy autonomy

Resilience in CPS refers to the capability of complex systems, integrating connected cyber and physical components to deliver services that can justifiably be trusted when facing changes [12].

The concept of resilience originates in diverse disciplines, including psychology, in which it is defined ‘as the process of adapting well in the face of adversity, trauma, tragedy, threats or significant sources of stress—such as family and relationship problems, serious health problems, or workplace and financial stressors' [13]. The word resilience is also used in physics and mechanical engineering, representing ‘the capability of a strained body to recovering its size and shape after deformation caused especially by compressive stress’ [14]. More recently, the concept has been associated with CPS, the latter defined as complex entities integrating connected cyber and physical components, which are used in several domains, including intelligent transportation and Industry 4.0 [15].

In the field of computer-based systems, the most accredited definition of resilience builds on the definition of dependability, the latter being defined as ‘the capability to deliver services that can justifiably be trusted’ [16]. Trust refers to dependability attributes that are reliability, availability, maintainability, safety, integrity and confidentiality, whereas the subset of attributes represented by availability, integrity and confidentiality is also collectively known as ‘security’. Threats can be faults (i.e. causes of errors), errors (i.e. wrong system state due to faults) and failures (i.e. incorrect service originating from errors). Several other classifications of faults are possible depending on their domain (i.e. hardware versus software), persistence (i.e. transient versus permanent), etc. The most common technique to protect from faults is known as ‘fault-tolerance’. Compared to ‘dependable CPS’, ‘resilient CPS’ can deliver services that can justifiably be trusted even in the presence of changes such as system upgrades and evolution. It is worth mentioning that CPS are often associated with the concepts of distributed embedded systems and smart systems, with which they share aspects of complexity, autonomy and criticality [17]. The interest in resilient CPS has grown in the last years as witnessed by the numerous projects and publications on related topics [7].

Addressing resilience in CPS is extremely challenging due to size, heterogeneity, distribution and criticality, implying more complex analyses. Generally, engineers adopt model-based approaches for risk assessment together with a combination of hardware and software technologies to ensure both resilience-by-design and run-time monitoring for threat detection and management. When system complexity is high, modular and compositional approaches are needed based on multi-paradigm modelling, including abstraction, multi-formalism and meta-modelling [18]. Ensuring holistic resilience requires considering both intentional and unintentional threats, which are normally managed by different teams of cybersecurity and fault-tolerance experts. Therefore, the convergence between reliability, safety and security evaluation is one of the main issues to be faced when assessing resilience. Furthermore, there is no universally accepted indicator to measure resilience in CPS, as several metrics have been proposed in recent projects and papers [3], also addressing semantic models and ontologies [19]. Informal and diverse definitions can lead to ambiguities and misunderstandings with regard to threat coverage, system boundaries and involved dependability attributes. Since CPS can involve aspects of decision support and autonomy requiring AI, their resilience needs to be evaluated considering the issues of the so-called ‘trustworthy AI’ (i.e. ethical and robust AI) and ‘safe autonomy’, including topics such as explainable AI (XAI) and adversarial attacks to AI, which represent extremely current and open research fields [20]. Finally, since the definition of resilience stresses the importance of continuous change, in the system itself as well as in its environment, self-adaptation techniques are being investigated by researchers in order to achieve self-healing, according to the so-called MAPE-K (monitor–analyse–plan–execute over a shared knowledge) feedback loop [21]. That provides a structured approach to intelligent threat detection and management through optimal CPS reaction and reconfiguration [22]. In particular, how to perform response and recovery in CPS is a complex aspect to be considered in critical and constrained environments because guaranteeing a trusted response can be challenging in real environments [8].

## 3. Description of the conceptual framework

Due to the complexity of CPS, it is essential to depict a conceptual framework through a modular and multi-level stratified architecture enabling the paradigm of DT to incorporate run-time models supporting self-healing and trustworthy autonomy.

**Figure 1.**
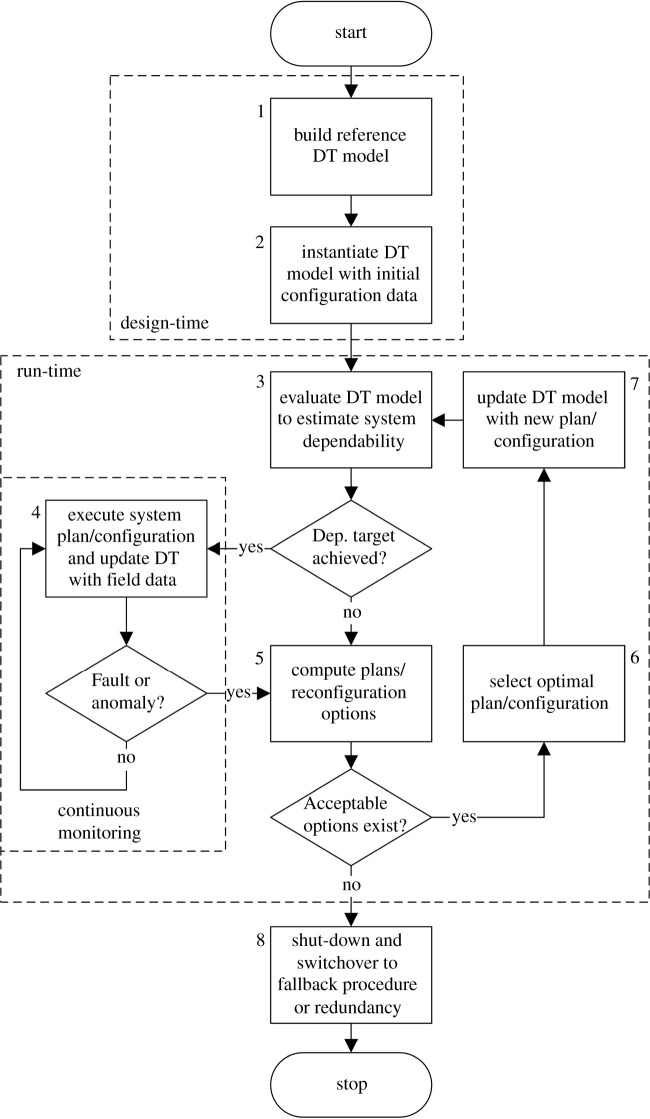
Flow chart describing the process of continuous monitoring and planning/reconfiguration through DT run-time models.

First of all, let us introduce the flow chart in figure 1, which summarizes the macroscopic phases required to build DT models and use them in order to monitor system dependability at run-time. Please consider that due to the high level of abstraction, the same approach can be applied to both single-autonomous components and complex systems-of-systems. The critical phase (labelled as number 3 in the flow chart) is dependability evaluation at run-time, which is followed—if the dependability target is achieved—by a continuous monitoring loop aimed at detecting faults (i.e. known causes of errors) or anomalies (i.e. any deviations from nominal behaviour, possibly indicating unknown faults and errors) before they can lead to system failures. Faults, errors and failures, as well as behavioural anomalies, can be collectively referred to as ‘threats’ to system dependability, according to most common taxonomies [16]. When those threats are detected, self-healing aims at finding the optimal reconfiguration or plan for reaction, depending on the nature of the threat: simple faults can be managed using legacy fault-tolerance mechanisms based on redundancy, fault-isolation and error correction within a single device; more complex, possibly intentional and strategic, cyber-physical threats may require adopting multi-step and multi-entity coordinated plans. ‘What-if’ analyses and predictions performed in real-time thanks to the run-time models inside DT will allow systems to autonomously take decisions on whether solutions are available to counteract threats and restore system operation, possibly with reduced capacity/performance, or rather a shutdown or switchover to another external—most likely human-controlled—system is required.

**Figure 2.**
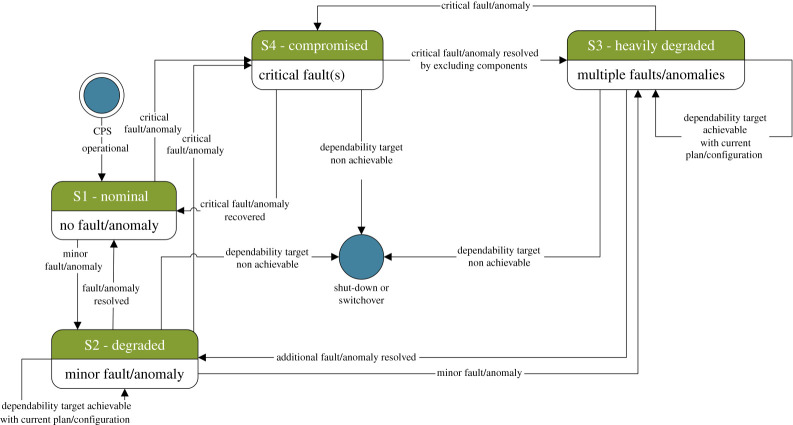
State chart describing the transitions among nominal, degraded and compromised states in self-healing CPS. (Online version in colour.)

Figure 2 shows another view of the same process by focusing on macroscopic states. Note that the diagram is not an exact representation of an abstract state-machine for any CPS, rather it represents an easy-to-understand example of system behaviour. It only features four states that summarize the most important situations for an autonomous CPS: in state 1, the system operates in nominal conditions with no faults or anomalies detected; state 2 represents a minor degradation in system operation with minimum or no impact on its capacity, in terms of both performance and tolerance to additional minor threats; state 3 represents a major degradation in system performance and/or capacity to counteract additional threats, but still guaranteeing vital functions such as safety-related ones; finally, in state 4a system is compromised due to a high number of minor faults or anomalies, or even due to a single critical fault. State 4 is necessarily transient because the system cannot safely continue its operation if no solution is promptly applied. From all degraded states, it is possibly to recover by applying appropriate fixes such as reconfigurations and response plans. Repairs can involve both hardware (e.g. switching to a backup physical component) and software (e.g. restarting processes, also known as ‘software rejuvenation’) [23]. Depending on safety-criticality, certain fixes such as SOTA (Software Over The Air) upgrades could not be possible, unless the whole process is fully validated according to reference international standards. Those standards often do not yet consider the new landscape of dependable intelligent systems operating in dynamic and evolving environments with many uncertainties to be faced; in such scenarios, missing prompt updates could be more harmful than not achieving full predictability. Nevertheless, run-time model checking approaches are possible for online validation of reconfiguration plans and recovery responses for adaptive CPS needing to comply with specific Safety Integrity Levels (e.g. see [23]).

**Figure 3.**
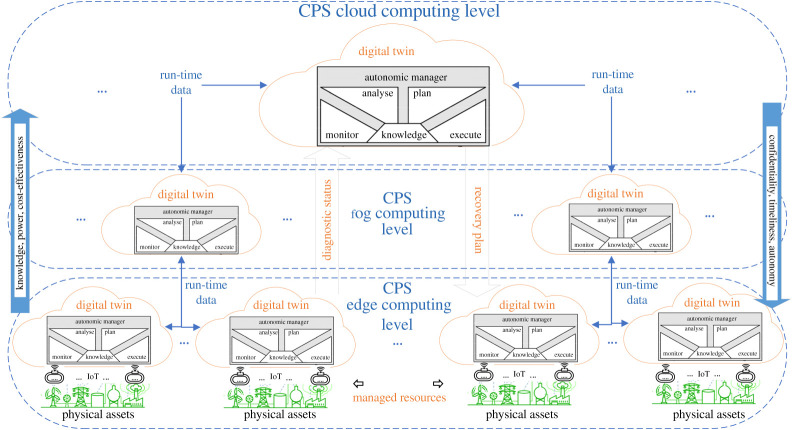
Conceptual architecture integrating the MAPE-K self-healing loop into DT at multiple levels of CPS. (Online version in colour.)

Based on the principles explained above, figure 3 depicts an overall conceptual architecture showing the possible location of DT hosting run-time models according to the MAPE-K control loop. The figure includes the three main levels that nowadays constitute a common abstraction on CPS modelling:

— **Edge**, i.e. field device level (e.g. smart sensor/actuator).
— **Fog**, i.e. local area network / metropolitan area network (e.g. gateway or local server).
— **Cloud**, i.e. wide area network (e.g. datacentre in a remote geographical location).

Depending on where the DT is located, a trade-off is achieved among:

— **Knowledge**, i.e. available information, which is likely to increase as the level moves to the cloud, due to the collection of bigger amounts of usable data coming from multiple devices as well as from similar systems installed worldwide; all that information can be used in data-driven approaches such as deep learning for predictive maintenance, which work better when DT are located at the cloud level.
— **Power**, i.e. computing and storage capacity, which is much higher in the cloud due to the scalability of datacentres, compared to the possibly very limited power of cheap and constrained edge devices.
— **Cost-effectiveness**, i.e. reduction of implementation costs, which is normally possible when adopting external cloud services allowing for resource optimization.
— **Confidentiality**, i.e. protecting data from unauthorized access (and possible corruption), which is easier at edge and fog levels where less transmission means is used in controlled environments with limited usage of open networks and remote storage.
— **Timeliness**, i.e. ensuring quick (e.g. real-time and predictable) local response, which is expected to be better at lower levels due to reduced transmission latency, compared to possible delays and retransmissions due to packet losses over external wide area networks.
— **Autonomy**, i.e. capacity to react to threats and other unknown situations locally with the (limited) information available even when Internet connection is unavailable, which is clearly a requirement that can be fulfilled only if DT are implemented at the edge computing level.

As depicted in figure 3, knowledge about the physical assets is typically collected using IoT sensing devices. Therefore, an abstract digital representation of the real world would be available depending on the nature, number and position of those devices. The same holds for any physical countermeasures that would be orchestrated by the DT in response to threats. In semi-autonomous systems, humans can also be considered part of the system as information sources, through structured interfaces and feedbacks, as well as actuators, through step-by-step guided responses. Whether humans or other devices are acting as sensors, it is essential to account for faults and uncertainties through appropriate trust models.

Figure 3 provides a hierarchical representation with highly redundant DT that are implemented at each level. Clearly, in real-world implementations, a trade-off should be achieved among all the factors mentioned above, considering the criticality and specific requirements of each application, as well as cost considerations.

Considering the MAPE-K loop included in each DT, run-time models are essentially fed by the available *knowledge* and used in the *analysis* and *planning* phases, e.g. to evaluate *what-if* options in building the optimal response plan. *Knowledge* includes all relevant information about the system, environment, threats and countermeasures, such as Bayesian representations [22] and troubleshooting information retrieved from open-data repositories (e.g., machine-readable product manuals [23]). In real-world implementations, DT could be added-on or rather built-in the CPS; all the specific implementation aspects such as using standard communication protocols rather than ad-hoc ones, service-oriented architectures (SOA) middleware [24], etc. are not in the scope of this overview, although some hints for future research will be provided in the next section.

It is important to observe that a hierarchical approach allows for better management of complexity and optimization of resources, with a distributed computing approach using available knowledge and power at each level. In such a way, local autonomy would be possible even though the connection is lost to the higher levels, or hard real-time constraints would not allow a response from the cloud. In turn, this could lead to a suboptimal reaction due to limited knowledge and power. In addition to higher fault-tolerance due to redundancy, a full hierarchical approach also supports separation of concerns, with local models elaborating a different subset of information and providing the results of those elaborations to more abstract—but possibly much bigger in terms of input data—models at higher levels. An example of this could be the software monitoring of a connected self-driving car, in which a prompt action might be required at a local (i.e. edge computing) level to recover from the crash of a software process requiring the switchover to a backup module or even to manual operation, depending on the achievable dependability target; at cloud level, specific details to support fault recovery such as full log file data might be omitted to save resources, while it would be important to understand whether the same type of failure has happened to similar vehicles in similar conditions, in order to plan for software patching or return to the workshop. Also, information about the successful adoption of response actions at the edge level should be propagated through the cloud in order to be used in similar devices in analogous situations. In such a view, DT hierarchy is associated with model abstraction and modularity rather than pure redundancy and enables federated multi-simulation among DT for predictive analytics [25].

Another aspect worth mentioning is that although the main scope of the concept is to support self-healing, which could be interpreted as an extremization of fault-tolerance in complex CPS, the approach also allows for fault-avoidance [16], due to the autonomic controller implementing the MAPE-K look in the DT possibly being able to predict the future evolution of CPS behaviour and react accordingly in order to prevent threats. In fact, in this application the autonomic controller can be thought of as a resilience controller over the managed system also ensuring safe autonomy whenever appropriate and feasible.

## 4. Challenges in anomaly detection with digital twins

The framework presented in the previous section is based on the capability of DT to detect anomalies by analysing CPS events and diagnostic data generated at the edge level. Anomaly detection is a broad topic which can be defined in several ways. According to the definition given in one pioneering work on the subject [26]:

### (a) Anomaly detection refers to the problems of finding patterns in data thatdo not conform to expected behaviour

For instance, collective anomalies can be found in inputs that consist of datasets where multiple instances of the dataset considered together convey information regarding an anomaly. Vice-versa, point anomalies are anomalies found in datasets where single instances may turn out to be anomalies.

Since anomaly detection works with datasets, data mining techniques are used together with other techniques, which consider multiple instances at a time, such as

— Classification-based anomaly detection techniques, which can be applied whenever enough labelled data is available.
— Clustering-based anomaly detection techniques, which identify ‘normal’ regions where data must reside in order to not consider the system as misbehaving.
— Statistical anomaly detection techniques, where a statistical model is fit to ‘normal’ data: when run-time system data does not fit with reasonable confidence the statistical model, the system is misbehaving.

A more specific and recent survey on IoT anomaly detection has been provided in reference [27]. Each IoT device can be abstracted as a process communicating with other processes in the network, thus configuring a highly heterogeneous distributed system, which requires ad-hoc middleware solutions to enable communication. The survey highlights that IoT devices are constrained in terms of resources, which make them more prone to being corrupted and cause security problems and behavioural anomalies. The survey also explores the possibility of adopting techniques for IoT intrusion detection, namely the class of anomaly detection methods, where the nominal behaviour is modelled, and anomalies are detected through identifying deviations from normal behaviour using run-time data.

More generally, the massive use of digital technologies has brought to the definition of the *Internet of events*, where an enormous amount of data is collected [28]. *Data science* is a broad field that covers all the disciplines which deal with or contribute to the extraction of value from data collected from digital applications. Applying data science when inspecting data is key to recovering new information, data patterns, models and solutions to recurrent problems. Data science also couples with another science, which is concerned with the analysis of behavioural processes of applications: process science. *Process science* has, as its main goal, the design, development, validation, maintenance and enhancement of process models, i.e. models that describe the behaviour of all kinds of applications (for example, business applications). The discipline known as *process mining* is the result of coupling data science with process science. Process mining has been shown to be a valuable alternative to detect and predict faults or anomalies in IoT applications. In particular, it has three intended uses:

— **Process discovery**, where, starting from event data, new process models can be discovered (play-in).
— **Conformance check**, where, starting from event data and one or more existing process models, data can be replayed on the model(s) to verify the conformance of the behaviour of the application with the considered process models.
— **Enhancement**, where, starting from event data and one or more existing process models, data can be replayed on the model(s) to carry out performance and dependability analyses.

Process mining is also a broad discipline: when discovering a process model, and hence using a certain notation, different perspectives may be captured. One of these is the control-flow perspective, where the ordering of activities is found and recurrent patterns are captured by a model; one notation suitable for this perspective is the *Petri net* modelling notation. A broad class of algorithms, with the only input being the (pre-processed) event log obtained by the events collected, can extract Petri nets; one example of such algorithms is the α-algorithm.

In the field of process mining applied to CPS resilience, reference [29] proposes a framework to detect anomalies from event data coming from the edge, based on process discovery from available data. After a model is retrieved, additional data are collected and each trace, i.e. each sequence of related activities in data, is replayed on the model. If differences are detected when replaying the event log on the model, then a *fitness* parameter is determined in order to compare it with a specified threshold. When a single trace in the event log has too many differences when replayed on the model and thus a low fitness parameter is extracted, then the system is classified as misbehaving.

In addition to formal models needed to recognize threats, anomaly detection also requires specific architectures to collect and vehiculate relevant data. In the field of IoT, a layered approach can be used to decompose functional aspects. In particular, a layer known as *middleware* can be used as an application enablement platform, i.e. to integrate external applications and ensure requirements such as interoperability, persistence and analytics, context awareness, resource and event handling, etc. In fact, IoT devices are normally unable to perform resource-intensive tasks. To that end, some researchers have introduced the concept of the *cloud of things*, extending the IoT with cloud computing services [30]. As already mentioned in the previous section and highlighted in reference [30], using cloud services to enhance CPS resilience via DT requires taking into account issues related to bandwidth, connection unavailability, latency, data validity, security, etc. The authors of [30] propose a set of requirements that a service-oriented middleware must satisfy to provide self-management mechanisms such as self-configuration, self-healing and self-optimization of service providers, which also support IoT anomaly detection. Additional edge-fog-cloud computing architectural paradigms for anomaly detection can be found in [31,32], where a detailed explanation of specific functionalities of each platform and a description of the specifications that each device should have to be employed in a certain layer are explored and properly motivated.

## 5. Conclusion

When Jean-Claude Laprie associated the concept of resilience to computer systems for the first time in a publication dated 2008 [12], he probably could not imagine the huge importance that the term would have gained in the following decades. That pioneering work, together with the previous dependability taxonomies and fault-tolerant architectures, served to settle the main pillars for all modern concepts of self-healing CPS and trustworthy autonomy.

In this paper, we have associated the concepts of resilience, self-healing and trustworthy autonomy to the paradigm of DT through run-time models embedded in the MAPE-K loop of autonomic computing.

We have provided an overview of the main concepts and their interrelations as well as some reference abstract models and architectures for continuous CPS monitoring for faults and anomalies using DT and self-healing mechanisms. We believe that current approaches to self-healing and trustworthy autonomy should conform as much as possible to widely accepted taxonomies and reference architectures in order to ensure systematicity and verifiability. That is a way to manage complexity and criticality, which otherwise would diverge due to the growing challenges of resilience assessment of large, distributed and heterogeneous CPS in the presence of changes, evolutions and uncertainties, both in the environment and the systems themselves. Those aspects are nowadays crucial, and it is therefore essential to develop new approaches to improve CPS resilience that are modular, compositional and scalable. We have shown that the combination of the DT paradigm with autonomic computing through the MAPE-K loop and related run-time models could be an effective route towards the achievement of that paramount objective. Regarding the current implementation challenges of such a paradigm, we have highlighted recent related research based on service-orientation and multi-layered edge-fog-cloud computing platforms. Although the main objective is to achieve full autonomy in future CPS, it is worth mentioning that partial or incremental autonomy in a ‘human-in-the-loop’ fashion would also be an option, with DT acting as decision support systems, whenever legal requirements still oblige to human supervision. Given the current state of the art, one promising future research direction should aim at data-driven anomaly detection through process mining, in order to enhance prediction capabilities by leveraging the huge potential of big data analytics [33].

## Data accessibility

This article does not contain any additional data.
